# Supplementary material for: Analysis of Centranthera grandiflora Benth Transcriptome Explores Genes of Catalpol, Acteoside and Azafrin Biosynthesis
Source: Int J Mol Sci. 2019 Nov 29;20(23):6034. doi: 10.3390/ijms20236034 (PMC6928798; doi:10.3390/ijms20236034)
Supplement: Supplementary file 1 [file ijms-20-06034-s001.pdf]

Supplementary Figures:

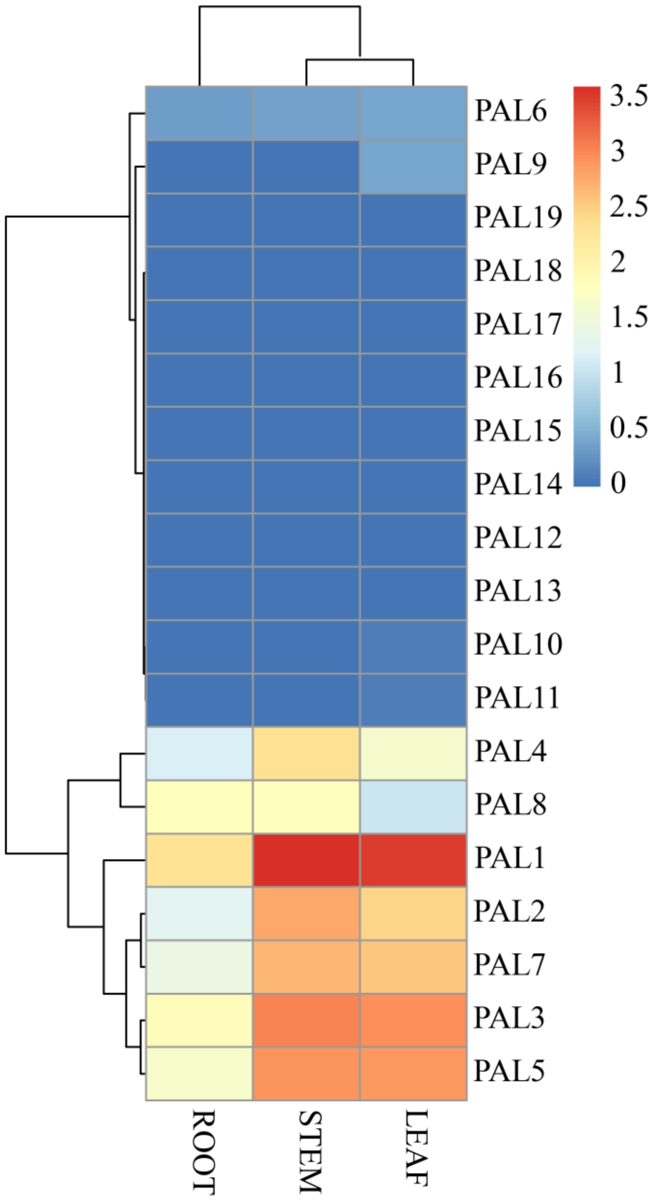

Figure S1. Expression of *PAL* genes in the putative pathway of acteoside biosynthesis across three tissues in *C. grandiflora* benth. The expression level is the sum of all the unigenes for each gene, and $\log_{10}$(sum(FPKM)+1) was used to plot the heatmap. Candidate *PAL* unigenes were selected according to the annotation. Abbreviation: PAL, phenylalanine ammonia-lyase.

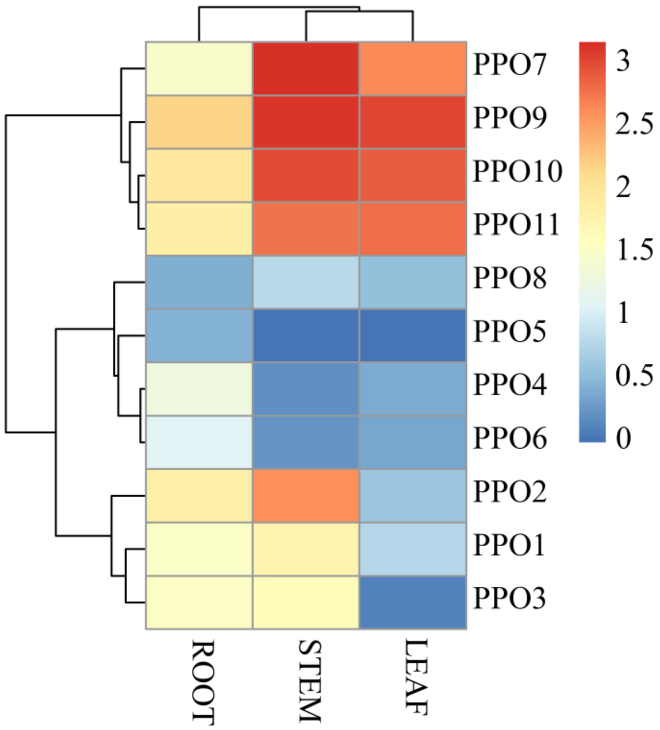

Figure S2. Expression of *PPO* genes in the putative pathway of acteoside biosynthesis across three tissues in *C. grandiflora* benth. The expression level is the sum of all the unigenes for each gene, and $\log_{10}$(sum(FPKM)+1) was used to plot the heatmap. Candidate *PAL* unigenes were selected according to the annotation. Abbreviation: PPO, polyphenol oxidase.

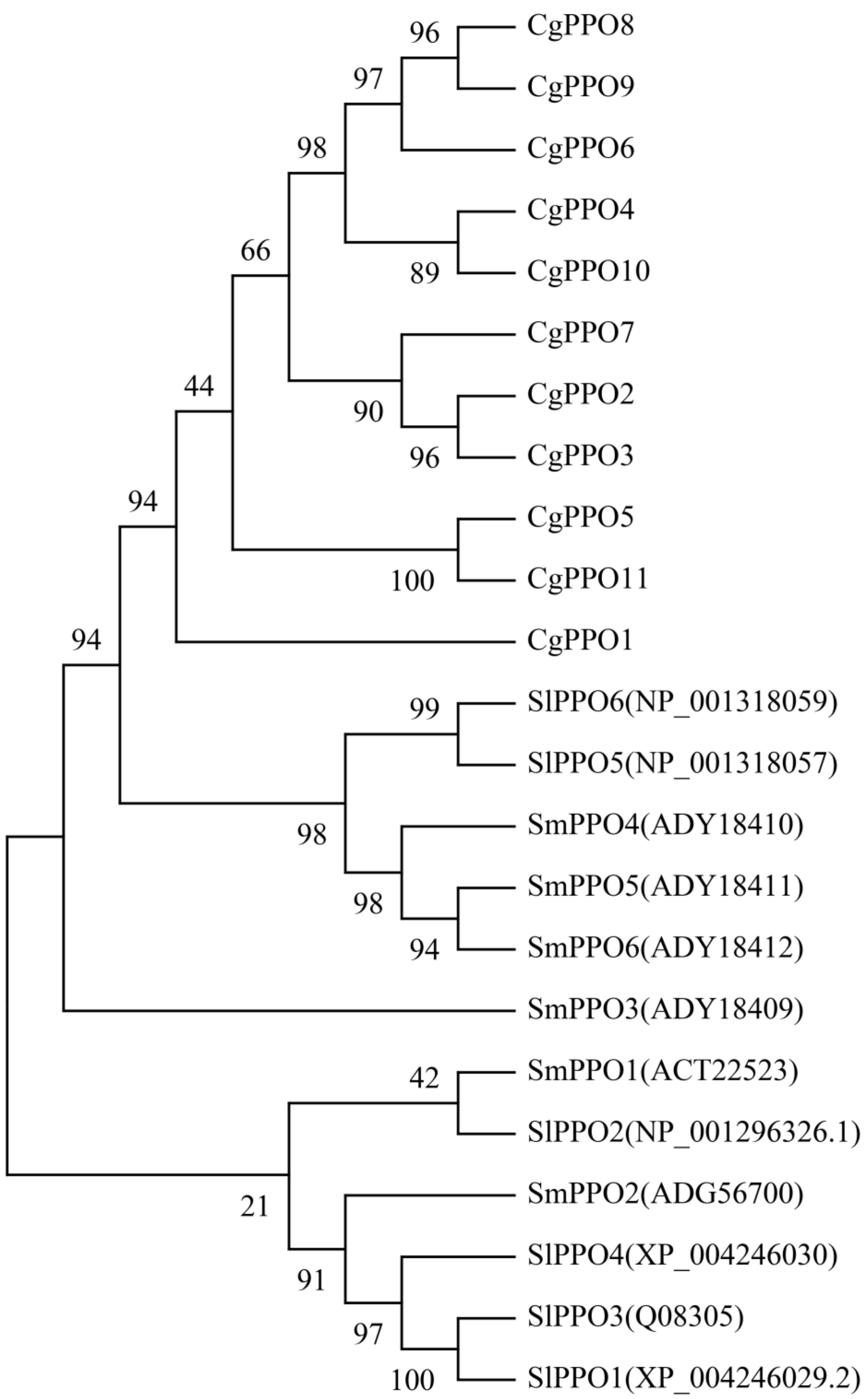

Figure S3. Phylogenetic analysis and expression level of PPOs from *C. grandiflora* Benth. Amino acid sequences were aligned using the ClustalX2 program, and evolutionary distances were calculated using phyML software with the Maximum Likelihood statistical method.

Note: Supplementary tables S1-S14 are too big (277 Mb) to upload at present.

Now the fourteen tables are uploaded into Zenodo website, please download them using this link:

https://zenodo.org/record/3408408#.XdYpF9OTccB

You can find in the website:

zenodo

Search

Upload Communities

Log in Sign up

November 21, 2019

Journal article Open Access

Analysis of Centranthera grandiflora benth Transcriptome Explores Genes of Catalpol, Acteoside and Azafrin Biosynthesis

Zhang, Xiaodong

Supplementary tables for the article "Analysis of ***Centranthera grandiflora*** benth Transcriptome Explores Genes of Catalpol, Acteoside and Azafrin Biosynthesis" in International Journal of Molecular Sciences.

Files (289.9 MB)

Name	Size	
Supplementary tables.rar	289.9 MB	Download

md5:a3525ba39cbb9f6f9601da50876bc4ca

Beta

Citations 0

Show only: Literature (0) Dataset (0) Software (0) Unknown (0)

Citations to this version

Search

0 views
0 downloads
See more details...

Indexed in
OpenAIRE

Publication date:
November 21, 2019

DOI:
DOI 10.5281/zenodo.3408408

Keyword(s):
Centranthera grandiflora benth; transcriptome; catalpol biosynthesis; acteoside biosynthesis; azafrin biosynthesis

License (for files):
Creative Commons Attribution 4.0 International
